# Cobalt starvation affects multiple cellular processes in Desulfofundulus kuznetsovii TPOSR during alcohol oxidation

**DOI:** 10.1099/mgen.0.001771

**Published:** 2026-09-03

**Authors:** Lukas Friedeheim, Sjef Boeren, Irene Sánchez-Andrea, Alfons J.M. Stams, Diana Z. Sousa

**Affiliations:** 1 Laboratory of Microbiology, Wageningen University & Research, Wageningen, The Netherlands; 2 Laboratory of Biochemistry, Wageningen University & Research, Wageningen, The Netherlands; 3 Science and Technology School, IE University, Segovia, Spain

**Keywords:** alcohol dehydrogenase, cobalt starvation, comparative proteomics, *Desulfofundulus kuznetsovii*, methanol metabolism

## Abstract

Cobalt influences the methanol metabolism of *Desulfofundulus kuznetsovii* TPOSR, specifically by modulating the activity of one of its alcohol dehydrogenases (ADH), Adh1. However, the effects of cobalt on the broader proteome of strain TPOSR, as well as the utilization of alcohols besides methanol, remain unexplored. Here, proteomic analyses of strain TPOSR grown with and without cobalt on different alcohol substrates show that cobalt starvation impacts multiple cellular processes, including cobalamin biosynthesis, iron-sulphur cluster assembly and, most prominently, energy metabolism as indicated by altered abundances of hydrogenases and NAD(P)-dependent oxidoreductases. Despite the presence of six ADH-encoding genes in the genome, Adh1 is the dominant ADH during growth not only on methanol but also on several primary alcohols and diols (ethanol, 1-propanol, 1,2-propanediol, 1,3-propanediol, butanol, pentanol and heptanol). Enzymatic assays with purified Adh1 confirm activity with these substrates, except 1,3-propanediol, and show no activity toward secondary alcohols (2-propanol and 2-butanol). Comparative proteomics analyses of other sulphate-reducing microorganisms (SRMs), namely *Desulfofundulus australicum* and *Solidesulfovibrio carbinolicus*, further indicate that methanol and ethanol oxidation in SRMs is mediated by a single ADH/AOR pair. Together, these findings highlight the central role of cobalt in alcohol metabolism in strain TPOSR and identify conserved ADH/AOR enzymes as promising candidates for biotechnological applications.

**Impact Statement**

Sulfate-reducing microorganisms (SRMs) play important roles in global carbon and sulfur cycling, but their alcohol metabolism remains poorly understood. We identify a dominant alcohol dehydrogenase, Adh1, capable of oxidizing a broad range of primary alcohols in *Desulfofundulus kuznetsovii* TPOSR, and show that cobalt availability strongly influences alcohol metabolism and cellular energy processes. Comparative analyses suggest that a single ADH/AOR pair may mediate methanol and ethanol oxidation across diverse SRMs. These findings advance our understanding of alcohol metabolism and trace metal requirements in SRMs, with potential implications for biotechnology.

## Data Summary

The data generated or analyzed during this study are included in this publication and its Supplementary Information files. The mass spectrometry proteomics data have been deposited in the ProteomeXchange Consortium via the PRIDE partner repository under the following dataset identifiers: PXD047013 for the *D. kuznetsovii* TPOSR data from dataset 1, and PXD058525 for the *D. australicum*, *S. carbinolicus*, and *D. kuznetsovii* TPOSR data from dataset 2.

## Introduction

Anaerobic methanol oxidation has been extensively studied for methanogenic archaea and acetogenic bacteria, while the methanol metabolism of sulphate-reducing microbes (SRM) remained unexplored. In recent years, however, interest in this process has increased following the discovery of a unique co-expression of two methanol conversion pathways in *Desulfofundulus kuznetsovii* 17^T^ [1]: a cobalt-dependent methanol methyltransferase (MT), as typically employed by anaerobic acetogens [2, 3] and methanogens [4–6], as well as an alcohol dehydrogenase (ADH) pathway commonly used by aerobic methylotrophs [7, 8]. Proteomic analyses revealed that both pathways are active during growth on methanol in the presence of cobalt, while cobalt starvation specifically repressed the MT system [1]. However, the two-pathway setup is not universal among SRMs, not even within the species *D. kuznetsovii*. Recently, we characterized *D. kuznetsovii* TPOSR, which possesses an incomplete and non-functional MT operon, relying solely on the activity of its ADH, Adh1, for growth on methanol [9]. Despite the absence of the cobalt-dependent MT system, cobalt starvation still impaired the growth of strain TPOSR on methanol, and kinetic studies of Adh1, heterologously expressed in *Escherichia coli*, revealed reduced catalytic activity in the absence of cobalt [10]. While the reduced methanol turnover by Adh1 during cobalt starvation contributes to the impaired growth of strain TPOSR, a potential impact of cobalt deficiency on other metabolic processes remains to be investigated.

In addition to Adh1, the genome of strain TPOSR contains six genes encoding other ADHs [9], a feature that is widespread among SRMs. The presence of several ADH-encoding genes is observed not only in closely related Gram-positive species within the genus *Desulfofundulus*, such as *Desulfofundulus australicum* [11], but also in Gram-negative SRMs like *Solidesulfovibrio carbinolicus* [12]. *S. carbinolicus* can utilize methanol [12], and its methanol metabolism has been the focus of previous research [13]. Interestingly, when grown in the presence of both methanol and ethanol, *S. carbinolicus* consumed ethanol but was unable to subsequently oxidize methanol, despite being capable of methanol oxidation when provided as the sole substrate [13]. *D. australicum*, on the other hand, has been characterized as unable to metabolize methanol [11], despite harbouring an ADH/AOR encoding genes that are nearly identical to those highly abundant in the proteome of strain TPOSR during growth on methanol. This suggests that *D. australicum*, contrary to earlier reports, might have the capacity to utilize methanol.

In line with these observations, analyses of strain 17^T^ revealed the presence of six genes encoding ADHs that form distinct phylogenetic clusters, suggesting potential specialization for different substrates [1]. However, their abundance profiles were only assessed during growth on methanol and ethanol, under which conditions increased abundance of the same ADH was observed. Most ADHs in strain 17^T^ share high amino acid identity with homologous proteins in strain TPOSR. Notably, strain TPOSR has been demonstrated to utilize a broad range of alcohols beyond methanol and ethanol, including 1-propanol, 1,2-propanediol, 1,3-propanediol and butanol [9]. Nevertheless, it remains unclear whether oxidation of these substrates is mediated by distinct ADHs or whether the presence of multiple ADH-encoding genes reflects functional redundancy. The elevated methanol oxidation rates, together with the good thermal stability and oxygen tolerance of Adh1, have already been documented, establishing this enzyme as a promising candidate for methanol conversion [10]. However, a comprehensive understanding of the substrate scope of Adh1 is essential to explore its potential in applications beyond methanol conversion, such as in bioremediation [14] or for the production of biofuels, pharmaceuticals and fine chemicals [15–18].

This study aimed to investigate:

- ADH abundance profile in *D. australicum* and *S. carbinolicus* during growth on methanol, ethanol and lactate (control).
- ADH abundance profile in strain TPOSR during growth on longer chain alcohols, namely 1,2-propanediol, 1,3-propanediol, butanol, pentanol and heptanol.
- The broader effects of cobalt starvation on the proteome of TPOSR, beyond its effect on the methanol oxidation activity of Adh1.

The results suggest that *D. australicum*, *S. carbinolicus* and *D. kuznetsovii* strain TPOSR rely on a single ADH and AOR for the oxidation of both methanol and ethanol. Additionally, strain TPOSR utilizes the same ADH for the oxidation of 1-propanol, 1,2-propanediol, 1,3-propanediol, butanol, pentanol and heptanol; enzyme assays with heterologously expressed TPOSR Adh1 confirmed *in vitro* activity with all tested substrates except 1,3-propanediol. Finally, cobalt starvation resulted in differential abundance of proteins involved in cobalamin biosynthesis, iron-sulphur protein assembly, together with changes in energy metabolism reflected by altered abundances of hydrogenases and NAD(P)-dependent oxidoreductases.

## Methods

### Microorganisms and cultivation

*D. kuznetsovii* strain TPOSR (DSM 110707) was obtained from our culture collection at the Laboratory of Microbiology, Wageningen University and Research, The Netherlands. *D. australicum* (DSM 3852) and *S. carbinolicus* (DSM 11792) were purchased from the German Collection of Microorganisms and Cell Cultures (DSMZ, Braunschweig, Germany). Microorganisms were grown in serum bottles containing anoxic bicarbonate-buffered medium (19). The medium was dispensed into the serum bottles, which were subsequently sealed with rubber caps and aluminium cramps, and the headspace was flushed and pressurized with N_2_/CO_2_ [80/20% (vol./vol.), 1.7 atm]. Bottles with medium were autoclaved (121 °C, 20 min), after which carbon/electron source (i.e. methanol, ethanol, 1,2-propanediol, 1,3-propanediol, butanol, pentanol or heptanol), electron acceptor (sulphate) and trace elements and vitamins were added from separately sterilized anaerobic stock solutions. Cobalt-free medium was prepared by omitting cobalt and vitamin B12 (cyanocobalamin) in the trace elements and vitamin stock solutions. References in this manuscript to ‘cobalt-free’ always mean free of cobalt and vitamin B12. Bottles used for cobalt-free incubations were cleaned with an acidic solution (aqua regia – HNO_3_ and HCl in a molar ratio of 1/3) to remove any trace cobalt and other residues. We omitted the analytical quantification of residual cobalt since complete leaching of cobalt by aqua regia has been consistently documented [20, 21]. Bottles were inoculated with 1% (vol./vol.) of exponentially growing cultures. Cultures of *S. carbinolicus*, *D. kuznetsovii* TPOSR and *D. australicum* were incubated statically at 37 °C, 55 °C or 65 °C, respectively, in the dark. All used chemicals had analytical grade and were purchased from Sigma-Aldrich (St. Louis, MO).

### Analytical techniques

Microbial growth was tracked by measuring the optical density at 600 nm in a UV-1800 UV–Vis spectrophotometer (Shimadzu, Japan). Methanol concentration was measured using a GC-2010 (Shimadzu, Japan) equipped with a DB-WAX UI column (Agilent, Santa Clara, CA) with N_2_ as carrier gas at a constant pressure of 100 kPa and using a flame ionization detector at a temperature of 250 °C. Samples were injected using a headspace autosampler HS-20 (Shimadzu, Japan) with an oven set to 50 °C. Concentrations of other alcohols as well as intermediates and products of alcohol oxidation were quantified using a Shimadzu HPLC (Kyoto, Japan), equipped with a Shodex column (SH-1011) and UV/RID detectors. A flow rate of 1 ml min^−1^ was used with sulphuric acid (0.01 N) as mobile phase and column temperature set at 45 °C.

### Comparative proteomics

For proteome analysis strain, TPOSR was grown on methanol (20 mM), ethanol (20 mM), 1,2-propanediol (10 mM), 1,3-propanediol (10 mM), 1-butanol (5 mM), 1-pentanol (5 mM) and 1-heptanol (2 mM), with sulphate (20 mM) as electron acceptor. Growth was evaluated both in the presence and absence of cobalt and vitamin B12. *D. australicum* and *S. carbinolicus* were grown on methanol (20 mM) and ethanol (20 mM) with sulphate (20 mM) and addition of cobalt and vitamin B12. Bottles of *S. carbinolicus*, *D. kuznetsovii* TPOSR and *D. australicum* were incubated statically in the dark at 37 °C, 55 °C or 65 °C, respectively. As a non-alcohol control condition for proteomics studies, we analysed the proteome of the three microorganisms grown on lactate (20 mM) and sulphate (20 mM) in the presence of cobalt and vitamin B12, and for strain TPOSR also in absence thereof. All the assays were done in triplicate. For protein extraction, 50 ml of mid-exponential phase grown cells were harvested by centrifugation at 10,000*g* at 4 °C for 10 min. Pelleted cells were washed and subsequently resuspended in 100 mM Tris-HCl (pH 8). To disrupt cells, sonication was performed using a sonicator equipped with a MS72 microtip probe (Bandelin, Germany) at 50% amplitude for 15 s. Cell debris was removed by centrifugation at 10,000×*g* at 4 °C, and the supernatant was reduced using 15 mM DTT for 30 min at 45 °C. Proteins were denatured using 6 M urea, alkylated with 20 mM acrylamide (incubated at 21 °C for 30 min). The pH was adjusted to 7 using 10% trifluoroacetic acid (TFA). Protein aggregation capture was used to capture proteins by using 1-µm diameter magnetic carboxylate modified beads (GE Healthcare, Brøndby, Denmark) [22]. Acetonitrile 100% (two times current volume) was added to induce protein aggregation during 20 min shaking at room temperature. Beads were retained on the wall of the tube using magnets and washed with 70% ethanol in water and 100% acetonitrile. Remaining protein was digested overnight at room temperature by addition of 100 µl ammonium bicarbonate (50 mM) containing 0.5 µg sequencing-grade trypsin. The digested peptides were acidified to pH 3 using 10% TFA. Subsequently, the beads were retained, and the solution was filtered through a double-layer C8 extraction disc (CDS analytical, Oxford, PA). Filter-trapped peptides were eluted with a 50% acetonitrile and 0.1% formic acid solution and concentrated by evaporation of the organic solvent. Protein concentration was measured using the Pierce Bradford Protein Assay Kit (Thermo Fisher, Waltham, MA).

Per sample, 0.5 µg of protein was loaded directly onto a 0.10×250 mm ReproSil-Pur 120 C18-AQ 1.9 µm beads (Dr. Maisch, Germany, Ammerbuch-Entringen) analytical column (prepared in-house) at a constant pressure of 825 bar (flow rate of circa 600 nl min^−1^) with buffer (1% formic acid in water) and eluted at a flow of 0.5 µl min^−1^ with a 50 min linear gradient from 9% to 34% acetonitrile in water with 1% formic acid with a Thermo EASY nanoLC1000 (Thermo Fisher, Waltham, MA). An electrospray potential of 3.5 kV was applied directly to the eluent via a stainless-steel needle fitted into the waste line of a micro cross that was connected between the nLC and the analytical column. On the connected Orbitrap Exploris 480 (Thermo Fisher, Waltham, MA) MS and MSMS AGC targets were set to 300% and 100%, respectively, or maximum ion injection times of 50 ms (MS) and 30 ms (MSMS) were used. HCD fragmented (isolation width, 1.2 m z^−1^; 28% normalized collision energy) MSMS scans in a cycle time of 1.1 s. The most abundant 2–5+ charged peaks in the MS scan were recorded in data-dependent mode (resolution, 15,000; threshold, 2e4; 15 s exclusion duration for the selected m z^−1^ ±10 p.p.m.). LC-MS/MS runs with all MS/MS spectra obtained were analysed with MaxQuant 1.6.3.4 [23]. Peptides and proteins were considered reliable for further analysis when they had a false discovery rate (FDR) of <1% and when for a protein at least two peptides were identified, of which at least one was unique and one unmodified. Normalized label-free quantification (LFQ) MS1 intensities for each protein group were obtained from MaxQuant after summing all normalized peptide intensities for each protein group [23]. Log_10_ LFQ intensities were filtered and further analysed using Perseus version 1.6.2.1 [24]. Zero ‘Log LFQ’ values were replaced by values obtained by applying a normal distribution with a down-shift of 1.8 and a width of 0.3 (Perseus default values) to make sensible ratio calculations possible. Student’s t-tests were performed using the ‘LFQ intensity’ columns obtained with a significance level of *P*≤0.05 and S0=0.1. Fold change was expressed as the ratio of the averaged LFQ value of a protein across all replications between the two compared conditions [25].

### Experimental design

**Fig. 1.**
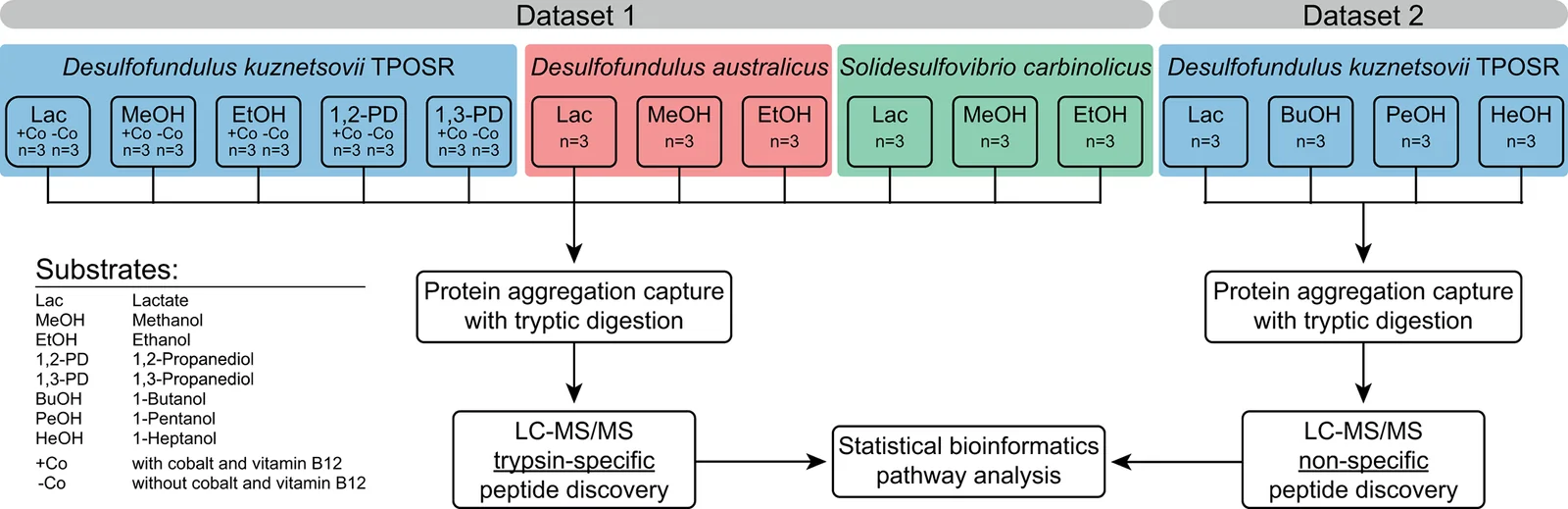
Experimental design and sample composition of the proteome analysis. Organisms and substrates used with substrate abbreviations explained in legend. The workflow of the sample preparation and analysis is outlined.

The proteome analysis was done for *D. kuznetsovii* TPOSR, *D. australicum* and *S. carbinolicus*. An overview of the experimental design is provided in Fig. 1. Dataset 1 analysed the proteome of strain TPOSR grown on lactate, methanol, ethanol, 1,2-propanediol and 1,3-propanediol, each in the presence and absence of cobalt and vitamin B12. In the same dataset, the proteome of *D. australicum* and *S. carbinolicus* grown on lactate, methanol and ethanol in the presence of cobalt and vitamin B12 was determined. In dataset 2, the proteome of strain TPOSR was measured during growth on lactate, butanol, pentanol and heptanol, in the presence of cobalt and vitamin B12. It is important to note that MS identification and quantification of one replicate from *D. australicum* grown on ethanol failed and was, therefore, analysed in duplicate. While dataset 1 was identified and quantified based on tryptic digestion, dataset 2 showed a significant percentage of non-specific protease activity likely resulting from protease contamination. Analysis of dataset 2 was conducted using the peptides resulting from non-specific digestion rather than trypsin digestion, resulting in a higher detection cutoff and reduced number of detected proteins when compared to dataset 1. This is reflected in the number of proteins per organism identified after strict filtering in at least one of the substrates: dataset 1: *D. kuznetsovii* TPOSR – 1728, *D. australicum* – 1427, *S. carbinolicus* – 2701; dataset 2: *D. kuznetsovii* TPOSR – 247.

### Plasmid construction and protein expression

Plasmid construction was performed as previously described by Friedeheim *et al.* [10]. In short, the DNA sequence coding for TPOSR Adh1 (G7K71_RS16190) was modified to contain an N-terminal TEV protease target sequence followed by a six-histidine repeat. The synthetic sequence was introduced into the pET26b expression plasmid and transformed into *E. coli* BL21. The protein was then purified according to the protocol described before [10]. In short, transformed cells were cultivated in selective LB (50 mg l^−1^ kanamycin) at 37 °C, and Adh1 expression was induced by adding IPTG to a final concentration of 0.2 mM, followed by overnight incubation. Cells were harvested, resuspended in binding buffer (50 mM HEPES pH 7.4, 300 mM NaCl, 20 mM imidazole) containing one tablet of cOmplete^TM^ protease inhibitor and sonicated on ice (half-inch probe, 25% amplitude, 10 min, 1 s ON/2 s OFF). The resulting supernatant was filtered (0.22 µm membrane filter) and purified by fast performance liquid chromatography using an ÄKTA Go Protein Purification system (GE Healthcare Life Sciences). The protein was loaded on a 5-ml His Trap™ HP column (GE Healthcare Life Sciences) pre-equilibrated with binding buffer, eluted with elution buffer (50 mM HEPES, pH 7.4, 300 mM NaCl, 500 mM imidazole) and subsequently desalted using a 5-ml HiTrap desalting column (Cytiva) pre-equilibrated with 50 mM HEPES, pH 7.4. Protein concentrations were determined using the Pierce™ BCA Protein Assay Kit (Thermo Fisher Scientific), with bovine gamma globulin as standard and stored at 4 °C.

### Enzymatic activity studies

To determine the enzymatic activity of the heterologously expressed Adh1 from strain TPOSR, the changes in the concentration of NADH were measured at a wavelength of 340 nm using a Synergy MX Multi-Mode Microplate Reader (BioTek). Kinetic assays for alcohol oxidation of TPOSR Adh1 were carried out in 50 mM BTP buffer in 96-well microplates (Greiner, 675101). The activity was measured at 55 °C, which was previously determined to be the optimal growth temperature of strain TPOSR and the temperature at which the highest activity of Adh1 was observed [10]. The concentration of Adh1 in the assays was 100 nM (4.28 µg µl^−1^) unless stated otherwise. Substrate stocks were freshly prepared, kept on ice and NAD^+^ concentrations were quantified spectrophotometrically. For the determination of the kinetic parameters, we used a saturating NAD^+^ concentration of 2 mM and alcohol concentrations from 0.02 to 10 mM. The kinetic parameters of the purified enzyme were determined using the software Dynafit (26). Model discrimination analyses were performed to determine which enzymatic mechanism best explains the experimental observations. The Dynafit script used during this analysis and the measured methanol oxidation rates are available in the supplemental information (Supplement 3).

## Results

Protein abundance profiles were obtained from strain TPOSR growing on lactate, methanol, ethanol, 1,2-propanediol and 1,3-propanediol in the presence and absence of cobalt and vitamin B12, and additionally during growth on lactate, butanol, pentanol and heptanol in the presence of cobalt and vitamin B12. Furthermore, protein abundance profiles were obtained from *D. australicum* and *S. carbinolicus* growing on lactate, methanol and ethanol in the presence of cobalt and vitamin B12. Variance in the LFQ values of protein abundance profiles across different substrates was visualized using principal component analysis for all the tested strains (Supplement 1, Fig. S1, available in the online Supplementary Material). The PCA indicates clear differences in protein abundance when comparing alcohol substrates with the lactate control, as well as among the different alcohol substrates. Furthermore, for strain TPOSR, PCA indicates distinct protein abundance profiles during growth with and without cobalt and vitamin B12 for all substrates except methanol, for which samples from cobalt-containing and cobalt-free conditions clustered closely.

### Strain TPOSR Adh1 is abundant during oxidation of several alcohols

**Fig. 2.**
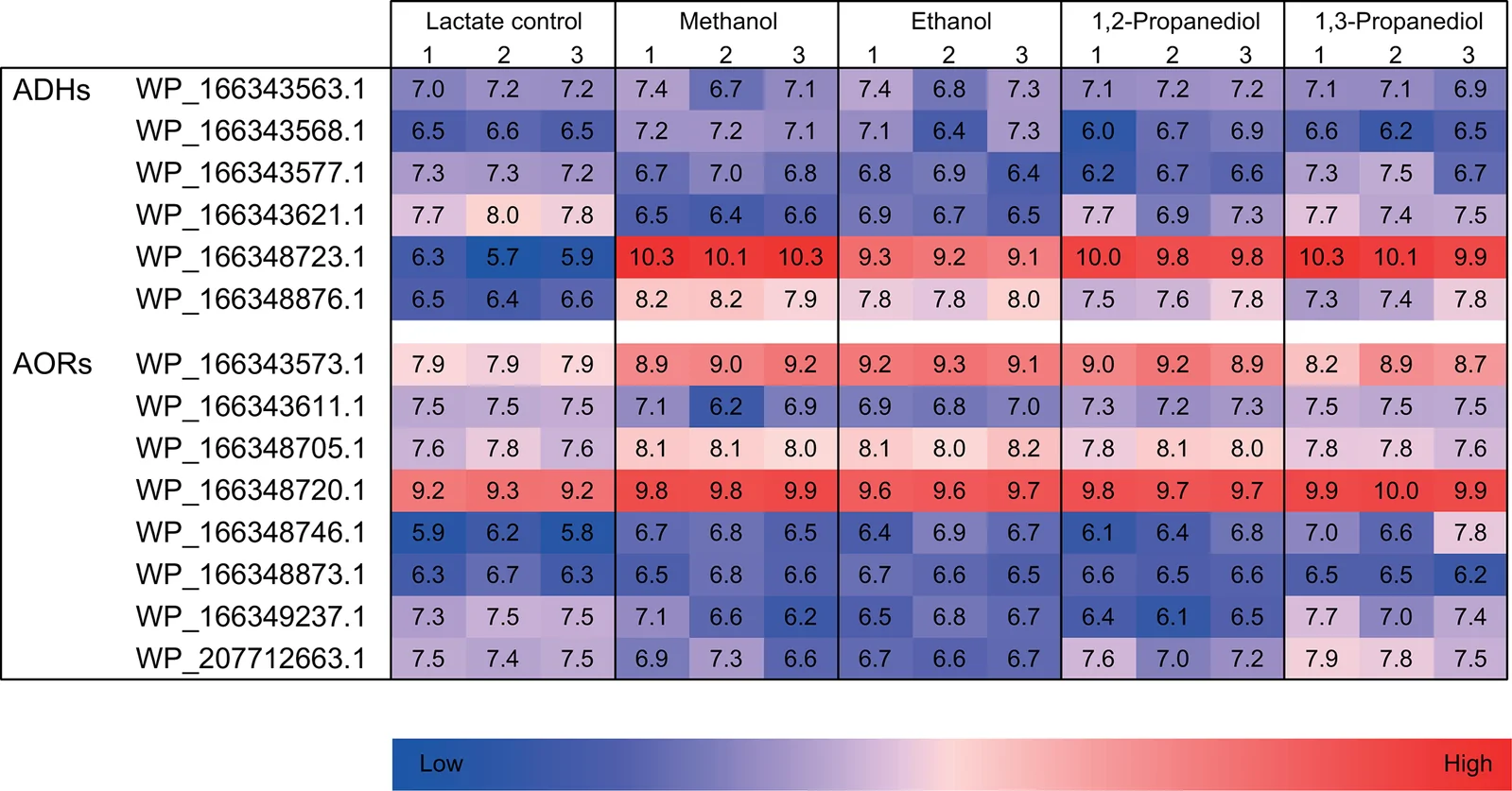
Abundance of ADHs (top) and AORs (bottom) in proteomes of strain TPOSR grown on different substrates (run 1). The heatmap represents the log_10_(LFQ) abundance of ADHs and AORs measured for indicated substrates. The highly abundant ADH/AOR pair is highlighted in bold.

The enzymes involved in the oxidation of methanol by strain TPOSR have been identified before (ADH, Adh1 - WP_166348723.1 and AOR - WP_166348720.1), and the activity of Adh1 with methanol was confirmed [10]. However, enzymes involved in the oxidation of longer alcohols and diols have not yet been determined. Here, we analysed the proteomes of strain TPOSR grown on methanol, ethanol, 1,2-propanediol, 1,3-propanediol, butanol, pentanol and heptanol to assess the abundances of their ADHs. The oxidation of all tested alcohols correlated with high abundances of the same ADH and AOR, namely WP_166348723.1 and WP_166348720.1, respectively (Fig. 2). The abundance of this ADH increased ∼1,500-fold during growth on ethanol (log_10_=9.2) and 45,000-fold during growth on pentanol (log_10_=10.7) relative to growth on lactate. In contrast, the adjacent AOR was already highly abundant during growth on lactate (log_10_=9.2) and increased only 3- to 20-fold during growth on alcohol substrates. High abundance of these ADH and AOR was also observed when strain TPOSR was grown on butanol, pentanol and heptanol (dataset 2), while the other ADHs were not detected in the proteome. In the proteome of cells grown on methanol, ethanol, 1,2-propanediol and 1,3-propanediol, a second ADH (WP_166348876.1) and an AOR (WP_166343573.1) were also detected at increased abundance; however, their abundances were substantially lower (log_10_=7.3–8.2 and log_10_=8.2–9.3, respectively). The other ADHs in the genome (WP_166343563.1, WP_166343568.1, WP_166343577.1 and WP_166343621.1) were either not differentially abundant or were slightly less abundant during growth on alcohol substrates compared to lactate. The same trend was observed for the abundance profiles of the other AORs.

### Strain TPOSR Adh1 exhibits *in vitro* activity with a broad range of alcohols

To determine whether the high abundance of strain TPOSR Adh1 during growth on the tested alcohols correlates with substrate utilization, we performed enzymatic assays using purified Adh1 to determine apparent kinetic parameters for the oxidation of various alcohols. The estimated kinetic parameters are summarized in Table 1. Consistent with the broad substrate utilization suggested by the proteome analysis, Adh1 catalysed the oxidation of methanol, ethanol, 1-propanol, 1,2-propanediol, 1-butanol and 1-pentanol, with ethanol being the preferred substrate. In contrast, no activity was detected with 1,3-propanediol. Even though TPOSR grows on 1,3-propanediol and Adh1/AOR are strongly upregulated under this condition, the lack of detectable Adh1 activity with 1,3-propanediol *in vitro* suggests that 1,3-propanediol oxidation may proceed via a different enzyme/system *in vivo* (with Adh1 induced for parallel alcohol metabolism or redox balancing), or that Adh1 requires physiological factors not reproduced in the purified-enzyme assay (e.g. partner proteins, cellular redox state, or intracellular conditions). Furthermore, no activity was observed with the secondary alcohols 2-propanol and 2-butanol.

**Table 1.** Apparent kinetic parameters of TPOSR Adh1 for the oxidation of given alcohols with NAD^+^. Reported values include confidence intervals at a 95% probability level

Substrate	*K*_M_ (mM) [CV]	*K*_cat_ (s^−1^) [CV]
Ethanol	0.47 [0.386, 0.569]	902.0 [833, 980]
1-Propanol	2.05 [0.946, 5.21]	433.0 [350, 596]
1,2-Propanediol	1.10 [0.112, 9.82]	29.7 [18.5, 62.7]
1-Butanol	1.21 [0.122, 18.1]	26.2 [16.4, 79.5]
1-Pentanol	0.250 [0.022, 21]	16.3 [10.1, 60.7]

### Cobalt starvation affects the proteome of strain TPOSR

**Fig. 3.**
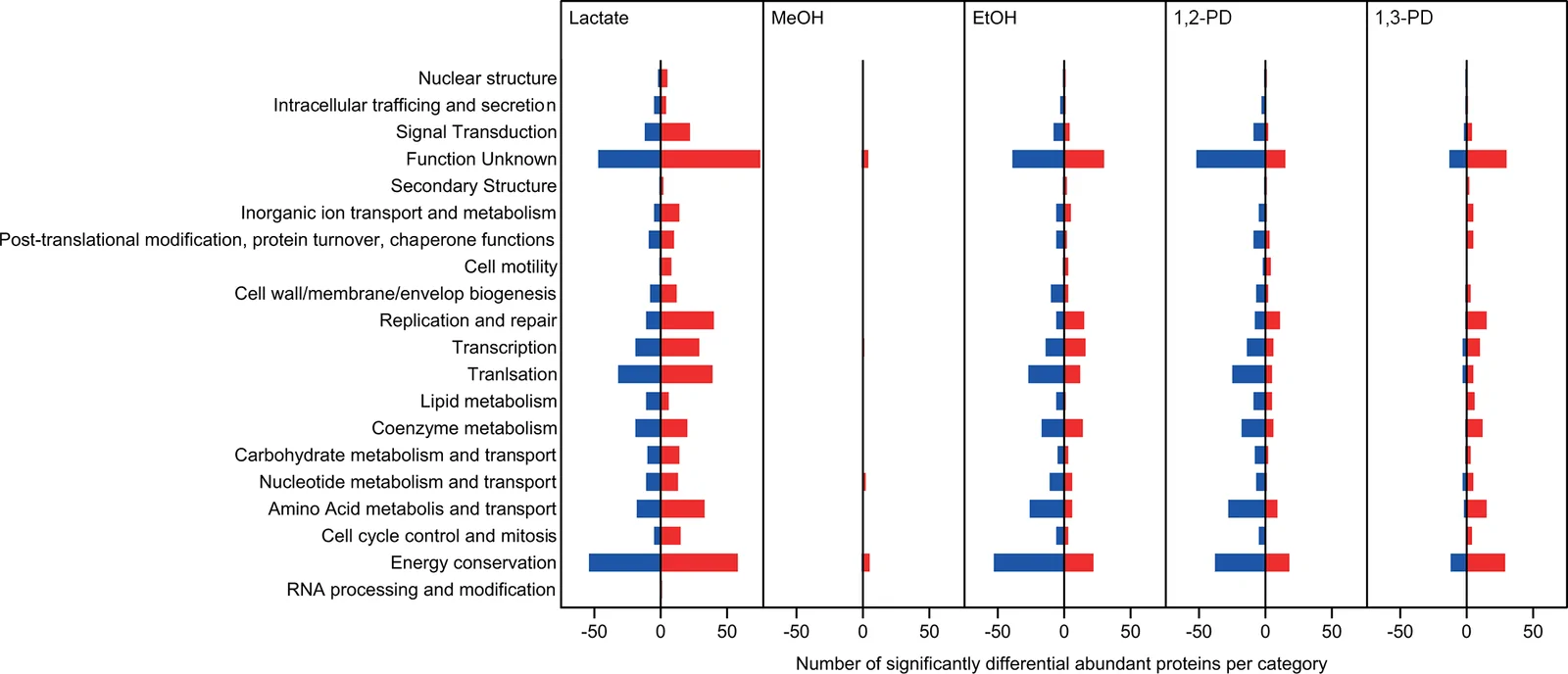
Differential protein abundance sorted by COGs for each of the tested substrates. Bars represent the change in protein abundance (positive/red=increased abundance, negative/blue=reduced abundance) from growth with cobalt to growth under cobalt starvation, considering only proteins with significantly differential abundance. The number adjacent to the bars indicates the number of proteins with higher abundance (positive) or with lower abundance (negative). The absence of a bar (and number) indicates that no proteins met the differential abundance criteria.

Previous research indicated that cobalt starvation inhibits the growth of strain TPOSR [9] and reduces the methanol oxidation rate of Adh1 [10]. However, the effects of cobalt starvation on the abundance profiles of other proteins have not been investigated. To assess the impact of cobalt availability beyond its role in the MT and ADH systems, we analysed the proteome of strain TPOSR grown in the presence and absence of cobalt and B12 with different alcohols, namely methanol, ethanol, 1,2-propanediol and 1,3-propanediol. While growth on methanol resulted in differential abundance of only a small number of proteins, substantially larger numbers of proteins were differentially abundant during growth on lactate (813), ethanol (450), 1,2-propanediol (379) and 1,3-propanediol (232). Classification of differentially abundant proteins according to clusters of orthologous genes (COGs) (Fig. 3) revealed that most changes were associated with energy conservation or proteins of unknown function. The remaining differentially abundant proteins were largely assigned to DNA replication, translation and transcription or to the metabolism and transport of amino acids and coenzymes.

### Cobalt starvation affects corrin/cobalamin biosynthesis, abundance of hydrogenases and nitrogen metabolism of strain TPOSR

**Fig. 4.**
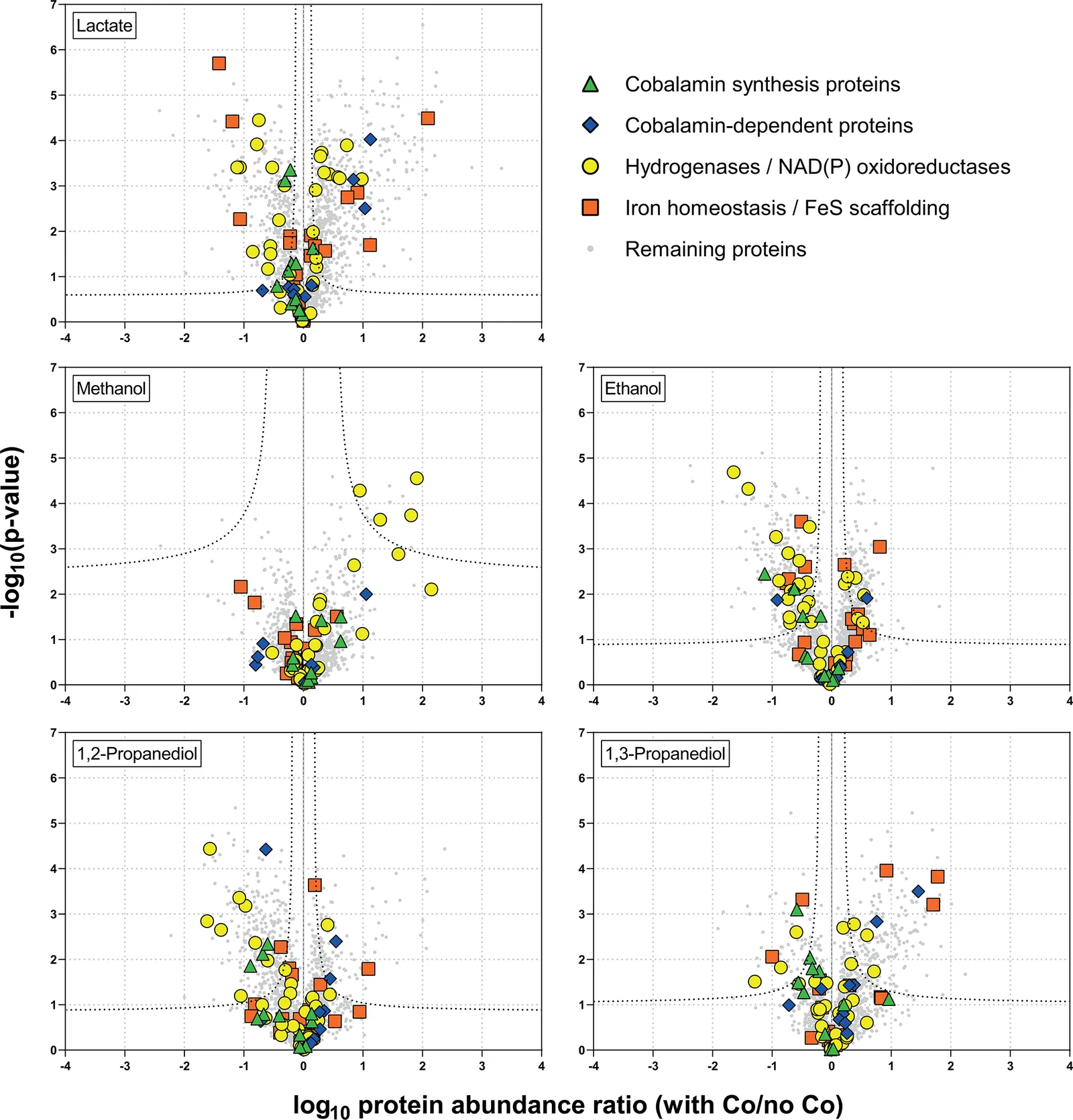
Comparative proteomics results reflecting differential protein abundance of cells grown on the given substrates, with and without supplementation of cobalt and vitamin B12. Data represent the analysis of three independent replicates. Colours represent protein groups (blue – corrin/cobalamin biosynthesis proteins, green – cobalamin-dependent proteins, yellow – hydrogenases and NAD(P) oxidoreductases and orange – iron homeostasis and iron-sulphur scaffolding proteins). The t-test (FDR=0.05, S0=0.1)-based significance level for each condition is indicated.

Proteins involved in coenzyme metabolism, particularly enzymes associated with the biosynthesis of the cobalt-dependent corrin and cobalamin cofactors, are influenced by cobalt availability [27]. In proteomes of strain TPOSR grown on lactate, ethanol, 1,2-propanediol and 1,3-propanediol, cobalt and vitamin B12 starvation, we observed consistently high abundance of proteins of the corrin and cobalamin biosynthesis pathways (Fig. 4). A similar upregulation of corrin and cobalamin biosynthesis proteins has been reported previously for the proteome of strain 17^T^ grown on methanol under cobalt starvation [1]. Sousa *et al.* [1] reported a significantly reduced abundance of methanol MT associated proteins in the proteome of strain 17^T^ during cobalt starvation. Although most components of this operon are absent in strain TPOSR, the remaining MtaA subunit and associated corrinoid-binding proteins likewise exhibited reduced abundance in the absence of cobalt. Other cobalamin-dependent enzymes essential for the reductive acetyl-CoA pathway include the corrinoid iron-sulphur protein, which serves as a methyl group carrier and the methionine synthase essential, which mediates tetrahydrofolate-dependent methyl transfer. In proteomes of TPOSR, the putative cobalamin methyl group carrier protein (WP_166342735.1, WP_166342737.1) remained largely unaffected by cobalt starvation, whereas proteins identified as methionine synthase-associated components (WP_166344411.1, WP_166344441.1 and WP_166344443.1) were detected at lower abundance in the absence of cobalt.

Besides the role of cobalt in cobalamin biosynthesis and cobalamin-dependent proteins, high cobalt concentrations have been shown to influence iron homeostasis. Inhibition of iron uptake by cobalt binding the ferric uptake regulator (FUR) has been reported [28]. In addition, cobalt can compete with iron during its integration in iron-sulphur containing proteins causing a reduction in enzyme activity [29]. Of the three proteins annotated as FUR in the TPOSR genome (WP_166343154.1, WP_166344672.1 and WP_166346669.1) only WP_166343154.1 and WP_166344672.1 were detected at low abundance and not affected by cobalt starvation. Iron transporters (WP_166344285.1 and WP_041282845.1) and scaffold proteins (WP_166347107.1) showed significantly lower abundance during cobalt starvation. At the same time, several iron-sulphur cluster proteins, particularly CoB-CoM heterodisulfide reductases, exhibited slightly increased abundance.

Cobalt starvation also affected energy metabolism, as reflected by changes in the abundance of TPOSR hydrogenases and NADH-quinone oxidoreductases (Fig. 3). During growth on lactate, ethanol, 1,2-propanediol and 1,3-propanediol, several proteins annotated as hydrogenases or NAD(P)H-quinone oxidoreductases were slightly more abundant under cobalt-starved conditions (Fig. 4). In contrast, during growth on methanol, cobalt starvation led to lower abundance of these enzymes. Consistent with this substrate-specific response, a hydrogenase (WP_166346439.1 – WP_166346444.1) that was abundant during growth on methanol and 1,3-propanediol in the presence of cobalt showed significantly decreased abundance during growth on methanol without cobalt (fold change=0.01), while its abundance during growth on 1,3-propanediol remained unaffected.

Finally, cobalt starvation was also associated with increased abundance of a sigma54-dependent Fis-family transcriptional regulator for all substrates except methanol. This regulator shows homology to nitric oxide reductase regulators (norR) from *Bacillota* species (MBP2641298.1). In addition, several proteins annotated with nitrite reductase-related (WP_166346187.1, WP_166345679.1 and WP_166344628.1) were more abundant under cobalt-starved conditions; however, corresponding metabolite data were not measured in the cultures.

### Alcohol oxidation resulted in acid excretion and hydrogen production

Whereas methanol was completely oxidized to CO_2_, longer chain alcohols underwent a two-step process involving alcohol and aldehyde oxidation, resulting in excretion of fatty acids despite the capacity of strain TPOSR to utilize these compounds. Oxidation of ethanol resulted in intermediary acetate accumulation. Similarly, metabolism of the longer-chain alcohols butanol, pentanol and heptanol resulted in accumulation of butanoate, pentanoate and heptanoate, respectively, with minor amounts of acetate and propionate. Oxidation of 1,2-propanediol resulted in the formation of lactaldehyde and lactate and oxidation of 1,3-propanediol led to excretion of 3-hydroxypropionate.

Despite acid excretion, increased abundance of beta-oxidation-related proteins was detected under all alcohol tests, indicating concurrent intracellular oxidation of fatty acids. These included acetyl-CoA acetyl transferases (WP_166344100.1), acyl-CoA dehydrogenase (WP_166344102.1, WP_279286272.1) and enoyl-CoA hydratase (WP_166344098.1). During growth on pentanol and heptanol, increased abundance of methylmalonyl-CoA mutase (WP_166342998.1) was additionally observed.

As a reference condition, growth of strain TPOSR on lactate resulted in acetate excretion and increased abundance of lactate metabolism-associated proteins, including lactate utilization protein (WP_166346300.1), LUD domain protein (WP_207712882.1) and lactate permease (WP_166346303.1). Similarly, during growth on 1,2-propanediol, accumulation of lactaldehyde and lactate was accompanied by increased abundance of the same lactate metabolism–associated proteins.

Alcohol oxidation in strain TPOSR is mediated by Adh1 and the adjacent AOR, the latter reducing ferredoxin, which can subsequently be reoxidized by hydrogenases to produce hydrogen. Consistent with this, several hydrogenases were more abundant during growth on alcohol substrates. Specifically, hydrogenases encoded by WP_166346439.1 – WP_166346444.1 were more abundant during growth on methanol and 1,3-propanediol, whereas a distinct hydrogenase (WP_166348776–WP_166348782) was more abundant during growth on ethanol, butanol, pentanol and heptanol. Increased abundance of hydrogenases during growth on alcohol substrates was associated with measurable hydrogen levels (not shown).

Finally, in all conditions we observed high abundance of proteins of the reductive acetyl-CoA pathway (formate–tetrahydrofolate ligase [EC:6.3.4.3], methylenetetrahydrofolate dehydrogenase [NADP^+^]/methenyltetrahydrofolate cyclohydrolase [EC:1.5.1.5 3.5.4.9], methenyltetrahydrofolate cyclohydrolase [EC:3.5.4.9], methylenetetrahydrofolate reductase [NADH] [EC:1.5.1.54] and acetyl-CoA decarbonylase/synthase [EC:2.3.1.169]) and of the sulphate reduction pathway (sulphate adenylyltransferase [EC:2.7.7.4], adenylylsulfate reductase [EC:1.8.99.2] and dissimilatory sulfite reductase [EC:1.8.1.22]). Interestingly, we detected a hypothetical protein (WP_013822538.1) with significantly increased abundance (fold change ~600) during growth on methanol; however, neither sequence-based nor structure-based prediction yielded sufficient information to hypothesize the function of WP_013822538.1.

### Methanol and ethanol oxidation in other sulphate-reducing microorganisms

To investigate the diversity and utilization patterns of ADHs in SRM, we extended our study to include *D. australicum* and *S. carbinolicus*. These microorganisms likely depend on ADHs for methanol and ethanol metabolism due to the absence of complete MT systems. *D. australicum* is a thermophilic, Gram-positive sulphate reducer closely related to strain TPOSR, while *S. carbinolicus* is mesophilic and Gram-negative, offering complementary perspectives across different environments and phylogenetic backgrounds.

*D. australicum* carries an incomplete MT operon lacking the catalytically required subunit B, but encodes seven ADH genes (WP_073162433.1, WP_073163824.1, WP_073164596.1, WP_073164733.1, WP_073165590.1 and WP_114362093.1, WP_114362093.1). Although *D. australicum* has been previously reported to be unable to grow on methanol [11], we observed growth in methanol-containing medium without addition of further substrates or additives. During growth on methanol and ethanol, a single ADH (WP_073163824.1) showed a significant increase in abundance (fold change=600). The adjacent AOR was already highly abundant during growth on lactate (log_₁₀_ = 9.7) and increased a further ~20 fold during growth on methanol and ethanol. Like strain TPOSR, constitutively high abundance of proteins associated with the reductive acetyl-CoA pathway and sulphate reduction was detected in *D. australicum*. Furthermore, hydrogenases homologous to the bifurcating/confurcating hydrogenases identified in strain TPOSR and 17^T^ were detected at high abundance during growth on methanol (WP_073162912.1 - WP_073162917.1) and ethanol (WP_073163866.1 - WP_073163869.1).

In contrast to the previously described members of the genus *Desulfofundulus*, *S. carbinolicus* does not have any of the genes of the MT operon in its genome and, therefore, methanol oxidation must follow the ADH route. We detected five genes encoding for ADHs (C3Y92_00795, C3Y92_01940, C3Y92_06170, C3Y92_10025 and C3Y92_16320) with C3Y92_16320 being most abundant during growth on methanol (fold change=30) and ethanol (fold change=4) compared to lactate. Unlike both strains of *D. kuznetsovii* and *D. australicum*, *S. carbinolicus* lacks an AOR adjacent to the highly abundant ADH; however, high abundance of two separately located AORs was detected across all substrates, namely C3Y92_00790 (log_10_=10.3–10.9) and C3Y92_10990 (log_10_=8.6–8.9). Interestingly, C3Y92_00790 is located next to an ADH (C3Y92_00795) that is detected at low abundance with all substrates (log_10_=7.2–7.6), while C3Y92_10990 is located adjacent to a NUDIX hydrolase that showed a slight increase in abundance during growth on methanol (fold change=4.5), but not during growth on ethanol. Again, we see abundance of NAD(P)H oxidoreductases during growth of *S. carbinolicus* on methanol and ethanol; however, hydrogen production was not assessed for this organism. Finally, also in *S. carbinolicus*, sulphate reduction-associated proteins show constitutive high abundance independent of the substrate used.

### The employed AORs share higher amino acid identity than the ADHs

**Fig. 5.**
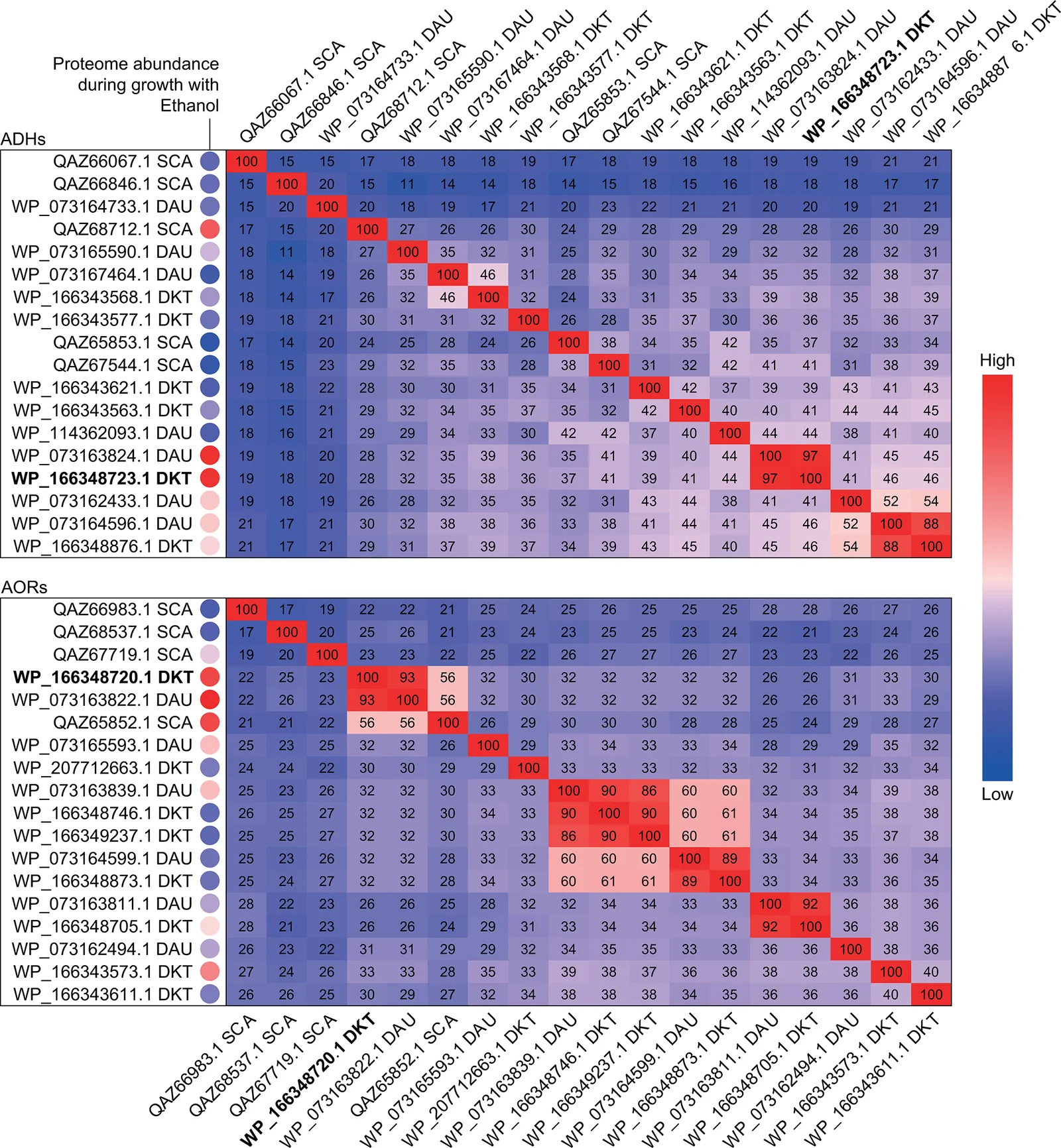
Amino acid identity matrix of ADHs (top) and AORs (bottom) from *D. kuznetsovii* TPOSR (DKT), *D. australicum* (DAU) and *S. carbinolicus* (SCA). Colours and data reflect pairwise percentage of amino acid identity. Coloured circles represent abundance of the respective enzyme in the proteome analysis of cells grown on ethanol.

We further investigated the amino acid similarities of ADHs and AORs of the studied strains (Fig. 5). *D. kuznetsovii* TPOSR and *D. australicum* share high ANI due to their close phylogenetic relation; accordingly, the employed ADHs of both strains shared 97% amino acid identity. In contrast, the abundant ADH of *S. carbinolicus* shared only 28–29% amino acid identity with the ADHs used by *D. australicum* and *D. kuznetsovii* strain TPOSR, despite the presence of two other ADHs with higher identity (37% and 42%). Compared to the ADHs, the abundant AORs used by the three strains showed substantially higher amino acid similarity. The AORs from strain TPOSR and *D. australicum* shared 93% identity, and the AOR from strain TPOSR shared 56% identity with the abundant AOR of *S. carbinolicus*.

## Discussion

The metabolism of longer-chain alcohols and diols in the sulphate reducer *D. kuznetsovii* TPOSR was investigated. Adh1 (WP_166348723.1), previously identified as the dominant ADH during methanol oxidation, was also highly abundant during growth on ethanol, 1,2-propanediol, 1,3-propanediol, butanol, pentanol and heptanol, indicating a broad substrate range for this enzyme. Consistent with this, the adjacent AOR (WP_166348720.1) was also abundant under all tested conditions, suggesting it catalyses the oxidation of the resulting aldehydes. A second AOR (WP_166343573.1) showed a lower increase in abundance, which may suggest functional redundancy. The metabolism of 1,2-propanediol typically involves a B12-dependent dehydratase [30], but no homologous genes were found in the genome of strain TPOSR, and proteome data instead suggest oxidation via the Adh1 and AOR. Metabolism of 1,3-propanediol is less studied; however, conversion of 1,3-propanediol to 3-hydroxypropionate was described for the sulphate reducer *Solidesulfovibrio alcoholivorans* (formerly *Desulfovibrio alcoholovorans*) [31]. In strain TPOSR, high abundance of Adh1 and AOR during growth on 1,3-propanediol is consistent with the formation of 3-hydroxypropionate.

The utilization of a single ADH/AOR pair for the oxidation of multiple alcohols raises questions about the evolutionary drivers of maintaining several ADH-encoding genes, which is not exclusive to *D. kuznetsovii*, but also found in other SRMs. Adh1 reduces NAD^+^, while AOR reduces ferredoxin, and both reduced cofactors can be reoxidized by bifurcating hydrogenases, proposed for the type strain 17^T^ [1]. The resulting hydrogen may contribute for energy conservation through a hydrogen-cycling mechanism [32]. Consistent with this, the abundant hydrogenases in strain TPOSR are homologous to the bifurcating hydrogenases of *Pelotomaculum thermopropionicum* [33] and strain 17^T^ [1]. As reported for strain 17^T^, there seems to be distinct hydrogenases employed during growth on methanol or ethanol. Furthermore, the hydrogenase abundant during growth on methanol was also abundant during growth on 1,3-propanediol, while the hydrogenase abundant during growth on ethanol was detected during growth on 1,2-propanediol, butanol, pentanol and heptanol.

Like strain TPOSR, proteome analyses of *D. australicum* and *S. carbinolicus* suggest that these organisms also utilize a single ADH/AOR for the metabolism of at least methanol and ethanol. In *S. carbinolicus*, when methanol and ethanol are supplied together, methanol is not consumed even after growth on ethanol has ceased [13]. This exclusive use of ethanol under mixed-substrate does not necessarily imply distinct enzymatic systems, and our proteome data instead suggests that oxidation of both alcohols is mediated by the same ADH. The lack of methanol oxidation after ethanol consumption could be a consequence of product inhibition caused by NADH, as previously demonstrated for TPOSR Adh1 [10]. In addition, during growth on methanol, *S. carbinolicus* exhibited increased abundance of a NUDIX hydrolase, which was not observed in either *D. kuznetsovii* [1] or *D. australicum*. This raises the possibility that the abundant ADH in *S. carbinolicus* may require an activator protein, as described for *Bacillus methanolicus* [8]. The fact that the NUDIX hydrolase increased in abundance only in cultures grown on methanol and not on ethanol suggests that such activation may be substrate- rather than enzyme-dependent. While NUDIX hydrolase was not abundant in the proteome of strain TPOSR, growth on methanol correlated with high abundance of a hypothetical protein (WP_013822538.1) that is conserved among the closely related species *Desulfofundulus salinum* [34] (96.26%, WP_121451600.1), *Desulfofundulus luciae* [35] (95.70%, WP_307403376.1) and *Desulfofundulus thermosubterraneus* [36] (94.12%, WP_072867588.1). Despite its methanol-associated abundance in strain TPOSR, *D. thermosubterraneus* and *D. luciae* are reported not to utilize methanol, whereas *D. salinum* does, leaving a potential role for this protein in methanol metabolism unresolved.

The broad substrate scope of TPOSR Adh1 suggested by proteomics results was corroborated by enzyme assays using the purified Adh1, while activity with methanol was reported before [10]. Our results suggest that Adh1 is capable of oxidation of primary alcohols, whereas the tested secondary alcohols – 2-propanol and 2-butanol – were not oxidized. This agrees with the previous observation that strain TPOSR does not grow on 2-propanol [9] and suggests that it is also unable to utilize 2-butanol as substrate. Adh1 showed comparable ethanol oxidation rates at high pH as other thermophilic ADHs from *Geobacillus thermodenitrificans* (*K*_cat_=638.7 s^−1^) [14] and *Flavobacterium frigidimaris* (*K*_cat_=474.9 s^−1^) [37]. The turnover numbers for 1-propanol, 1-butanol and 1-pentanol fall towards the upper range of existing data, suggesting that Adh1 holds significant potential for further exploration in biotechnological applications. Interestingly, although oxidation of 1,2-propanediol was observed, Adh1 did not oxidize 1,3-propanediol at a significant rate, despite its high abundance during the growth of strain TPOSR on this substrate. Given the previously documented high oxygen tolerance of Adh1 and the stimulating effect of cobalt ions on its activity, it was hypothesized that the heterologously expressed Adh1 purified from *E. coli* contains cobalt or nickel, whereas the native Adh1 in strain TPOSR likely incorporates iron [10]. While further investigation is needed, these differing cofactors could be the reason for the lack of activity with 1,3-propanediol of heterologous Adh1, even though it supported growth in its native host. Regardless, while cobalt has demonstrated a significant effect on the methanol oxidation of TPOSR Adh1 it remained a question whether it also influences the rest of the proteome.

Cobalt limitation affected cobalamin-related metabolism in strain TPOSR. Our data indicate decreased abundance of cobalamin-dependent proteins and a simultaneous increased abundance of proteins related to the biosynthesis of corrin/cobalamin in cobalt-free condition, although the differential abundances were small. Since the proteins involved in the synthesis of cobalamin are also involved in the salvage of these structures [38], the increased abundance could allow better retention of the remaining cobalt. The amount of cobalt retained in the culture and its change during the cobalt-free transfers was not analysed but could yield further insight. The intermediate of the cobalamin biosynthesis and salvage, cobinamide, has been shown to bind nitric oxide interacting with the nitrogen metabolism of the cell [39]. It is possible that this could be related to the increased abundances of the norR and the nitrite reductase. While we did not investigate the concentration of the associated metabolites of the nitrogen metabolism, this would be an ideal starting point for future research into the topic. Furthermore, cobalt starvation led to decreased abundance of iron transporters and Fe-S scaffolding proteins. It has been demonstrated that cobalt ions compete with iron ions for integration into iron-dependent proteins [28]. Therefore, the low cobalt concentration results in less competition and lower enzyme requirement. On the other hand, we observed a significant increase in abundance of several CoB-CoM heterodisulfide reductases which have been suggested to participate in ion pumping or electron bifurcation [40, 41]. Likewise, cobalt starvation increased abundance of several hydrogenases and NAD(P)-dependent oxidoreductases during growth on all substrates except methanol. How cobalt affects the abundance of hydrogenases remains puzzling, but a link to the generation of hydrogen is suggested. While hydrogenases are strongly metal-dependent, this generally refers to iron and nickel, and no cobalt-containing hydrogenase has been described to date. However, several synthetic cobalt complexes have been shown to exhibit hydrogenase-like functions [42], and the interaction of cobalt with the proton gradient, which appears to be particularly important during growth on alcohols, seems plausible.

In summary, this study delves further into the alcohol metabolism of SRMs showing a more widespread reliance of methanol and ethanol metabolism on a single ADH/AOR, despite abundance of several ADH and AOR encoding genes in the genomes. The Adh1 from *D. kuznetsovii* TPOSR shows high turnover rates with a broad range of alcohols, while the proteome-suggested activity with 1,2-propanediol remains to be investigated. Cobalt starvation appears to affect numerous cellular processes, most prominently the abundance of hydrogenases and other energy metabolism-related proteins, but the exact mechanism remains unclear. We, therefore, emphasize the importance of cobalt in the metabolism of methanol and further alcohols in SRMs and propose *D. kuznetsovii* as an ideal organism to further explore this, due to the unique genetic setup of the MT and ADH systems in the strains TPOSR and 17^T^.

## Supplementary material

10.1099/mgen.0.001771 Supplementary Material 1.

10.1099/mgen.0.001771 Supplementary Material 2.

## Abbreviations

ADH: alcohol dehydrogenases
BuOH: 1-Butanol
-Co: without cobalt and vitamin B12
+Co: with cobalt and vitamin B12
COG: clusters of orthologous gene
EtOH: ethanol
FDR: false discovery rate
FUR: ferric uptake regulator
HeOH: 1-Heptanol
Lac: lactate
LFQ: label free quantification
MeOH: methanol
MT: methyltransferase
norR: nitric oxide reductase regulators
1,2-PD: 1,2-Propanediol
1,3-PD: 1,3-Propanediol
PeOH: 1-Pentanol
SRM: sulphate-reducing microorganism
TFA: trifluoroacetic acid
