# Supplementary material for: Cobalt starvation affects multiple cellular processes in Desulfofundulus kuznetsovii TPOSR during alcohol oxidation
Source: Microb Genom. 2026 Sep 3;12(9):001771. doi: 10.1099/mgen.0.001771 (PMC13541210; doi:10.1099/mgen.0.001771)
Supplement: Supplementary file 1 [file mgen-12-01771-s001.pdf]

Supplemental information

PCA analysis of proteome measurements

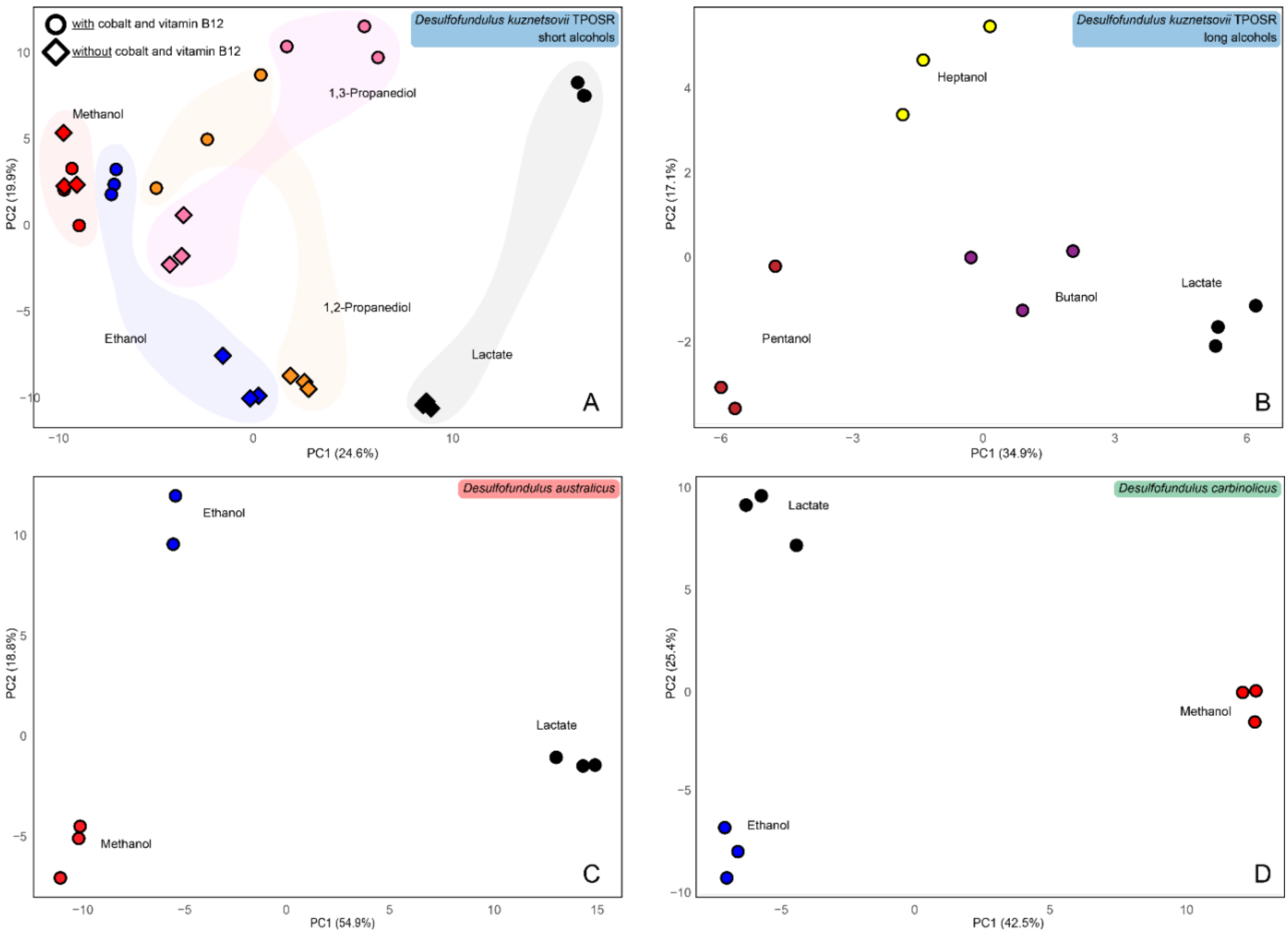

Figure S1. PCA analysis of proteome measurements from samples of A – TPOSR dataset 1 (samples from cells grown with cobalt and vitamin B12 in circles, without cobalt and vitamin B12 in diamonds), B – TPOSR dataset 2, C – *D. australicum* dataset 1 and D – *S. carbinolicus*. Proportions of explained variance of the principal components in each set are given in the axes labels, same colour represents same substrates and shaded areas (A) combine samples from same substrate grown with and without cobalt and vitamin B1.
